# Removal of Gall Stones—Ovarian Cyst—Spleen—Kidney and Ureter, En Masse

**Published:** 1896-07

**Authors:** Robert T. Morris

**Affiliations:** New York


### Clinical Lecture.

### REMOVAL OF GALL STONES—OVARIAN CYST—SPLEEN —KIDNEY AND URETER, EN MASSE.

By ROBERT T. MORRIS, M. D., New York.

### I.—REMOVAL OF GALL STONES.

THIS first patient has suffered for several months with pain in the region of the gall bladder, but has never had attacks of biliary colic and has never been jaundiced. He has been examined by several physicians, some of whom readily palpated the hard gall bladder, while others could not find it. We shall remove the gall stones by operation, believing that modern surgical methods are more to be respected than nature's ways in these cases.

My incision, about four inches long, exposes the margin of the liver, and we find the gall bladder firmly adherent to surrounding structures. Adhesions separated with a sweep of the finger, gauze is tucked about the gall bladder to keep things neat. I pass a loop of catgut through the wall of the gall bladder to serve as a handle, and then make an incision through which thirteen faceted gall stones are removed. Clear mucus flows from the incision. A little of this mucus is transferred to a culture tube, in order to determine what species of bacteria are present. We expect to find colon bacilli in the mucus or in the stones, because we have lately come to believe that gall stones are caused by colon bacilli, which climb up from the bowel into the bile ducts and cause a precipitation of cholesterin out of the bile. I find one small stone caught below one of the valves at the entrance to the cystic duct. Calculi

1. Delivered at the New York Post-Graduate Medical School, April 2, 1896.

are often caught at this point and it is difficult to move them up or down. This one is easily pushed through into the common duct, as it is a small one. If it were larger I would cut through the wall of the duct, take it out and then suture the duct. The incision in the gall bladder is sutured and the gall bladder allowed to assume its normal position. That makes the simplest operation. If the walls were thinner or if they were ulcerated, I would use the Murphy button and fasten gall bladder to the intestine, or would fasten the gall bladder to the abdominal wall and leave a fistula to close spontaneously. The abdominal wound is closed in tiers and the lesser omentum is arranged in a trough above a small niche drain, after sprinkling aristol along the line of the niche, so that if bile should leak out the niche will care for it. The trough of lesser omentum will guide it to the surface and the aristol coagulum will prevent leakage into the general peritoneal cavity. If I had cut and sutured a bile duct, the same technique would have been employed. Pure bile in the abdominal cavity does no harm, unless in very large amount, but if colon bacilli escape with it they may excite a peritonitis.

### II.—CYST OF LEFT OVARY.

The next patient is a nervous young woman. She has a cyst of the left ovary about as large as a butternut, but simple in its nature and evidently consisting of hydropsy of a Graafian vesicle. The cyst is shelled out, leaving the ovary. Most of us like to be conservative in our ovarian surgery nowadays, and we spare where some operators formerly wasted. The operation is done through a very small incision in the abdominal wall, and I am careful to make a nicely sutured wound because we are to avoid marring our patients. This patient has an adherent prepuce, so it is best to circumcise her after freeing the prepuce from the glands with the handle of the scalpel. Preputial adhesions have the same influence in girls that they have in boys, but they are much more common in girls.

### III.—REMOVAL OF SPLEEN.

The third patient has splenic leucocythemia and the lymphatic glands seem to be everywhere enlarged. He has one white blood corpuscle to every four red ones. The spleen is so enormous that it crowds the heart and lungs, and the patient breathes with great difficulty. He has been treated with red bone marrow for two months without benefit. It is always best to try red bone marrow treatment, because some of these cases are very much improved by it and occasionally one is apparently cured. I now make a median line incision, reaching from the sternum nearly to the pubes. Bowel is kept out of sight by sterilised towels. About two quarts of free peritoneal fluid escapes. I ligate five arteries running into the pulp of the spleen, cut the suspensory ligament from the diaphragm, and cut the spleen-stomach ligament. The spleen is separated from parietal connective tissue attachments with the flat hand and removed. On the scales we find that it weighs eight pounds and twelve ounces. The diaphragm does not contract. It has been distended so that it is paralysed and it feels more like a soft membrane than like a muscle. A hypodermatic injection of 1-12 grain of strychnine is given in the hope of stimulating the diaphragm into action. The patient has shown very little sign of shock and he has lost only the blood that was in the spleen and about a pint that has trickled out of torn parietal connective tissues, but as he cannot afford that loss we infuse two pints of physiological saline solution. This infusion apparatus is made up on the spot by putting a big hypodermic needle through a cork and sticking the cork into the rubber tube of the syringe. The whole thing is then boiled for five minutes for sterilisation. A transverse incision through the skin exposes one of the veins of the arm. The vein is pulled out of its bed and allowed to rest upon a needle passed under it. The hypodermic needle of the fountain syringe is passed into the vein and two pints of saline solution allowed to run into the circulation. The patient's pulse immediately becomes very full and strong. This is a very simple and safe way to make up for loss of blood. The abdominal wound is closed and you observe that, except for the dyspnea, the patient is in remarkably good condition.

### IV.—REMOVAL OF KIDNEY AND URETER EN MASSE.

I wish to report upon the case in which I removed the left kidney and ureter *en masse* a week ago. The specimen was sent to the laboratory and Dr. Brooks finds that the kidney was the seat of advanced interstitial nephritis. The ureter walls showed simple hypertrophy. As some members of the class were absent at the time of operation, I will state that this case was one in which the ureter had become obstructed by hyperplastic connective tissue resulting from a sigmoiditis, which at one time was probably perforative, as the patient had a general peritonitis beginning at that point. The patient after that suffered from ureteral colic. On palpation before operation I found that the left ureter was larger than the right one. Many physicians who have not had occasion to develop the sense of touch acutely, are not able to palpate ureters easily, but after a little practice they will find the ureter where it crosses the psoas muscle if they ask the patient to contract his psoas muscle a little, and having felt the ureter at that point, it is readily traced up to the kidney and down to the pelvis if the patient is not too fleshy. Almost anyone can palpate the free edge of the liver. Then, as his fingers become trained, he distinguishes colon from ileum. He later learns to find the normal appendix, the sub-peritoneal lymph glands, the iliac vessels, the ureter, the Fallopian tubes, and, in fact, feels more at ease with his fingers than with his eyes in abdominal work. In this case I made an incision from the left costo-quadrato angle to a point near the anterior superior spinous process of the ileum and extending down to the peritoneum. The kidney was brought into the incision and when its vessels were ligated and cut loose I used the kidney for a handle and easily stripped the ureter away from peritoneum until we reached the point of obstruction at the brim of the pelvis. It was exceedingly difficult to work out half an inch of ureter at that spot, but as soon as it was free the rest of the ureter was easily separated down to the trigone of the bladder. I broke off the ureter at the trigone with my finger nail, trusting to the valve action of the bladder wall for keeping the lumen closed afterward. I was pleased to find that no longer incision was required, as the one first made allowed the whole hand to be passed to the brim of the pelvis behind the peritoneum, and the finger in the pelvis readily came in contact with the bladder when the ureter was pulled upon sufficiently to stretch the bladder upward a little.

NOTE.—At date of writing, April 24, 1896. The spleen patient died on the night after the operation, apparently from asphyxia. The diaphragm would not perform its function in the respiratory act and the patient's dyspnea was distressing to behold. The other patients are practically well.

49 WEST 39TH STREET.

---

DOGTAIL SUTURES.—It is said that the tendons found in the tail of a dog make better sutures than either catgut or kangaroo tendon, when properly prepared in sublimate.—*Peoria Medical Journal.*

